# An experimental device for multi-directional somatosensory perturbation and its evaluation in a pilot psychophysical experiment

**DOI:** 10.1121/10.0001942

**Published:** 2020-09-18

**Authors:** Rintaro Ogane, Lynda Selila, Takayuki Ito

**Affiliations:** Université Grenoble Alpes, Centre National de la Recherche Scientifique, Institut Polytechnique de Grenoble, Laboratoire Grenoble Images Parole Signal Automatique, 38402, Saint Martin d'Hères Cedex, France rintaro.ogane@gipsa-lab.grenoble-inp.fr, lynda.selila@gipsa-lab.grenoble-inp.fr, takayuki.ito@gipsa-lab.grenoble-inp.fr

## Abstract

Somatosensory stimulation associated with facial skin deformation has been developed and efficiently applied in the study of speech production and speech perception. However, the technique is limited to a simplified unidirectional pattern of stimulation, and cannot adapt to realistic stimulation patterns related to multidimensional orofacial gestures. To overcome this issue, a new multi-actuator system is developed enabling one to synchronously deform the facial skin in multiple directions. The first prototype involves stimulation in two directions and its efficiency is evaluated using a temporal order judgement test involving vertical and horizontal facial skin stretches at the sides of the mouth.

## 1. Introduction

The somatosensory system is known to be an important component of speech motor control [e.g., Ghosh *et al.* (2010), Guenther and Vladusich (2012), and Perkell (2012)]. Its role has been long studied through the use of mechanical perturbations, either static or dynamic. Static mechanical perturbations such as bite-block (Lindblom *et al.*, 1979) or lip-tube (Savariaux *et al.*, 1995) experiments and repetitive exposure to dynamical mechanical perturbations (Tremblay *et al.*, 2003) provided evidence for compensatory adaptation and speech motor learning. Dynamic perturbations also enable one to study on-line control, as exemplified in the production of bilabial consonants, where immediate compensatory responses of the upper lip have been obtained with dynamic perturbations applied to the lower lip (Abbs and Gracco, 1983) or to the jaw (Folkins and Abbs, 1975; Kelso *et al.*, 1984).

In this framework, we have developed a dynamic somatosensory perturbation paradigm associated with facial skin deformation over the years (Ito *et al.*, 2009; Ito and Ostry, 2010, 2012). The rationale is that orofacial cutaneous receptors play a predominant role in providing kinesthetic information for speech motor control (Connor and Abbs, 1998; Johansson *et al.*, 1988). While other perturbation techniques generally affect the motor and somatosensory systems together, an important property of this method is to perturb only the somatosensory system, and hence to decouple the effects of these systems in the interpretation of experimental data. This allowed us to investigate the role of somatosensory inputs not only in speech production (Ito and Ostry, 2010), but also in speech perception (Ito *et al.*, 2009; Ogane *et al.*, 2020). However, the facial skin deformation technique has a severe practical limitation since the perturbation, using a single robotic actuator, can be applied only in one direction within a given experiment. In addition, due to the wire connection between the actuator and plastic tabs, the stretching force can only be applied to pull the skin. This strongly restricts experimental designs to specific combinations of speech stimuli and somatosensory stimulation. Ogane *et al.* (2020), in their study of the role of somatosensory inputs in a word segmentation task, showed that the effect of the somatosensory input was larger and more systematic when the vertical direction of skin deformation was coherent with the vertical direction of the corresponding articulatory movement in the involved speech material. Considering that the orofacial skin can actually be deformed in a variety of directions depending on speech utterances (Connor and Abbs, 1998; Vatikiotis-Bateson *et al.*, 1999), more realistic and varied perturbation patterns could hence be required to investigate speech production and perception mechanisms.

This paper introduces the framework of a new multi-directional facial skin deformation technique enabling us to pull the facial skin in multiple directions or at multiple positions at the same time, using multiple actuators. To provide a first implementation and experimental evaluation, we present a system in which both vertical and horizontal facial skin deformations are applied together on a single point. The system is then used for evaluation in a psychophysical experiment involving a temporal order judgement test where participants must decide whether the vertical stimulation occurs before or after the horizontal one. Finally, we discuss future applications and extensions of this new device.

## 2. Multi-directional skin-stretch perturbation system

### 2.1 Principle

**Fig. 1.**
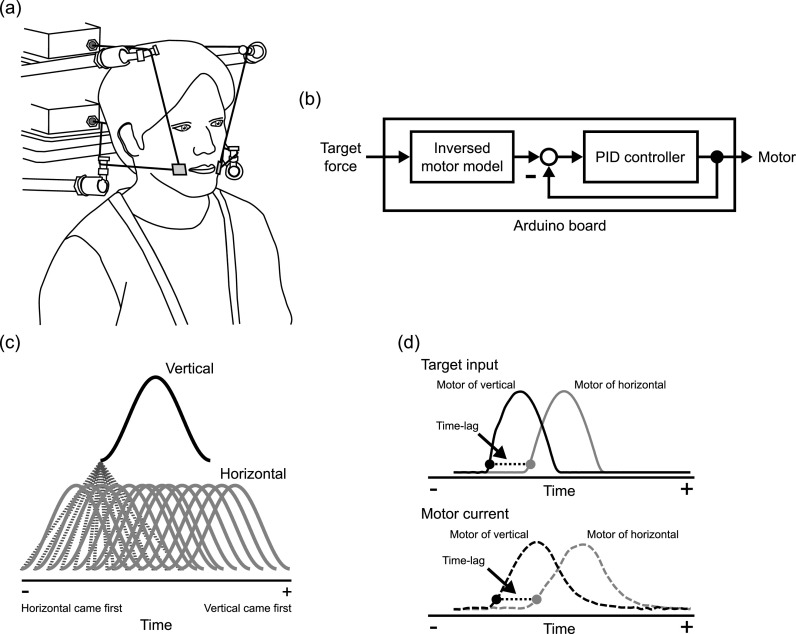
(a) One example of experimental configuration using a bi-directional skin stretch perturbation system, jointly applied on the two sides of the mouth. (b) Block diagram of force control for the motor. (c) Temporal pattern of vertical and horizontal stimulation with 14 values of time-lag. (d) Schematic illustration of the calculation of the time-lag between the two motors at the target input stage (top) and at the actual motor current stage (bottom). Black and gray lines represent the time course patterns, respectively, for the vertical and the horizontal motors. Dotted line represents the time-lag between the two components. This representative example corresponds to a case where the vertical motor leads the horizontal one.

We started from a basic configuration similar to the original system in Ito *et al.* (2009). In this system, skin stretch is delivered by an actuator device connected through thin wires to small plastic tabs (2 × 2 cm^2^) attached to the stimulation site by double-sided tape. To produce facial skin deformation in multiple directions simultaneously, we developed a system with multiple actuators connected to a single stimulus site. This system allows us to produce a variety of spatio-temporal patterns of facial skin deformation by controlling both the direction of the perturbation applied by each actuator, and the timing of each actuator separately. As a first step in the development of this new system, we developed a prototype with two actuators stretching the skin in two directions. Figure 1(a) shows one configuration of the proposed system, in which the stimulation is applied at both sides of the mouth simultaneously in the upward and backward directions.

### 2.2 System configuration

DC motors (Maxon 148867 motor, Switzerland) were used as stimulation actuators, instead of the robotic device used in the previous studies. The motors were controlled using a servo controller board (Arduino Due with Arduino Motor Shield board) connected to a laptop computer (Operating System: Windows 7 64 bit, CPU 2.60 GHz, 16.0 GB memory) through a USB. One servo board allows one to connect and control two motors. Since these motors and control boards are rather cheap (typically 400 euros for a motor and 70 euros for a board compared to approximately 20 000 euros for the robot), this could be easily extended to more than two motors in further applications.

While position control is easily applied for the control of a motor, we rather adopted force control to ensure a consistent amplitude of the force, but not the position, across trials, conditions, and participants for psychophysiological experiments. Force control is achieved by controlling motor current input and exploiting the known electromagnetic relationship between current input and force in this kind of motor. Figure 1(b) shows a block diagram of the force control system. The target force specified in input is converted into current using an inversed motor model. The converted target current is compared with the actual motor current, measured in the Arduino Motor Shield board. The resulting error between target and motor currents is then processed by a proportional–integral–derivative (PID) controller and the resultant signal drives the motor. This was programmed in simulink with available software packages (matlab Support Package for Arduino Hardware and simulink Support Package for Arduino Hardware, versions 18.2.0). PID gains were adjusted in unloaded conditions (that is, with no connection between the motors and an external device). This program runs at a 100-Hz sampling frequency—hence the timing of motor control can be controlled with a precision of 10 ms—and it enables one to simultaneously record target input and motor current for further data analysis.

## 3. Evaluation in a behavioral experiment

To examine the feasibility and precision of the proposed system for a psychophysical experiment, we carried out a temporal order judgement test, in which the perceived temporal order of vertical and horizontal skin stretches was evaluated. We expected that participants would correctly and easily identify the order when the time-lag between the two stimulations was sufficiently large, whereas the performance would decrease with small time-lags.

### 3.1 Method

Three participants were tested for this task. The protocol was approved by the Comité d'Ethique pour la Recherche, Grenoble Alpes (CERGA: Avis-2018–12-11–4).

The task was to identify whether the facial skin stimulation in the vertical direction came first or second, by pressing a key on the keyboard as quickly as possible. The applied skin stretch consisted in a sinusoidal pulse at 6 Hz (≈ 167 ms duration) with a peak amplitude at 2 N, as used in Ogane *et al.* (2019, 2020). Fourteen time-lag values (−120, −100, −80, −60, −40, −20, −10, 10, 20, 40, 60, 80, 100, 120 ms), where positive values correspond to cases where the vertical stimulation was applied earlier than the horizontal one [Fig. 1(c)], were tested in pseudo random order. 210 responses (14 time-lags × 15 responses) were recorded in total for each participant.

### 3.2 Data analysis

We evaluated two aspects: control performance of the developed system and perceptual performance of the participants' judgement. For control performance, we examined whether the system correctly produced the intended time-lag between the two motors in the test, by analyzing target inputs and motor currents, all transformed in force through the linear relationship between current and force. The data were up-sampled to 1000 Hz by linear interpolation and smoothing. The time-lag was calculated as the difference of onsets between the two motors at each stage (target input and actual motor response) [Fig. 1(d)]. The onsets in each signal were detected as the instant when the signal exceeded 10% of maximum amplitude. The obtained time-lag was averaged for 15 trials in each time-lag condition. A Pearson's product-moment correlation coefficient was applied to assess the adequate relationship between intended delay at the input target stage and actual delay at the motor stage.

For perceptual performance, we computed for each participant and each time-lag the probability for “vertical stimulation first” response. We then fitted a psychometric function to the distribution of averaged probabilities for each participant and estimated the 50% judgement cross-over and the just-noticeable difference (JND), provided by half the difference in time-lag between 25% and 75% judgement probabilities.

The data and analysis programs for correlation analysis of time-lag and for perceptual performance are provided as supplementary material.^1^

## 4. Results

### 4.1 Device performance for the control of time-lag between the two actuators

**Fig. 2.**
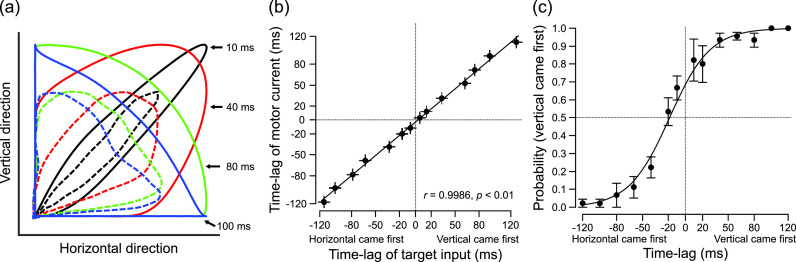
(Color online) (a) Spatial trajectories resulting from the combined action of the two motors obtained in one representative trial in 10, 40, 80, and 100 ms time-lag conditions. Solid lines represent the trajectories for target input and dashed lines for motor current. Each color corresponds to each time lag condition. (b) Relationship between time-lags for target inputs and for actual motor currents, together with the corresponding regression line and correlation value. Each dot represents the value averaged across 15 trials. Error bars represent standard errors. (c) Judgement probabilities averaged over the three participants and the estimated psychometric function in the temporal order judgement test. Each dot indicates the averaged probability and error bars represent standard error across the participants.

Figure 2(a) shows spatial trajectories of skin stretch perturbation resulting from the combined action of the two motors for four representative time-lags (10, 40, 80, and 100 ms). The solid lines represent the target input and the dashed lines represent the motor current. Beginning with target trajectories in solid lines, we check that for the smallest time-lag (in black), the trajectory is close to a diagonal movement, and when the time-lag is close to a half-cycle difference (≈83 ms), the trajectories are close to a rectangle triangle (in green and blue). Comparing trajectories between target inputs and actual motor currents, it appears that the intended spatial patterns of stimulation were successfully produced for each time-lag, although the amplitudes of the actual motor current were smaller than the target inputs because of facial skin stiffness. The correlation between target time-lags and actual ones shows a clear linear relationship [*r* = 0.9986, *p* < 0.01, Fig. 2(b)]. These results confirm that this new system can control accurately time-lags between the two directions of somatosensory stimulation. This enables to produce a variety of spatio-temporal patterns of somatosensory stimulation, which provides an adequate basis for assessing the psychophysical test of temporal order judgement in Sec. 4.2.

### 4.2 Psychophysical performance in the temporal order judgement test

Figure 2(c) shows judgement probabilities averaged across the three participants together with the estimated psychometric function. As expected, judgment probabilities follow gradual changes from 0.0 to 1.0 along the time-lag scale. Averaged judgement cross-over, −18.37 (±7.21 S.E.) ms, is marginally different from zero (*p* = 0.063, one-tailed one sample t-test). The JND is evaluated at 25.15 (±2.24 S.E.) ms.

## 5. Discussion

The current study proposed a framework for developing a new multi-directional somatosensory perturbation system associated with facial skin deformation. Based on this framework, we developed a two-directional somatosensory perturbation system with two actuators. The developed system showed an adequate quality of performance, enabling us to control accurately time-lags between two actuators and to generate accordingly various patterns of skin deformation, which was assessed in a somatosensory stimulation paradigm. In a psychophysiological test concerning temporal-order judgement, the participants perceived adequately the temporal order of two sequential orofacial stimuli as we expected. These results suggest that the proposed framework could overcome potential technical limitations of somatosensory perturbation experiments previously observed and discussed [e.g., Ogane *et al.* (2020)], and that the system can generate more realistic patterns of somatosensory stimulation related to human articulatory movements. The current framework can thus improve our investigation of the somatosensory role in human functions, particularly in speech perception, speech production, and their relationships in speech communication.

In the first stage of our study, our technical evaluation of the developed system showed that the control performance appeared rather accurate. First, the control of the onset time-lag between the two actuators is adequately precise [see Fig. 2(b)]. Given that the JND in temporal order judgement amounts to about 25 ms as observed in the psychophysical evaluation of the system, a precision around 10 ms in time-lag control should be adequate for testing temporal sensitivity of the somatosensory system. Concerning the control of amplitude, it appears that the amplitudes of the two actuators are also efficiently controlled considering the relatively neat diagonal trajectory produced when the two motors worked at the same time [corresponding to small onset time-lag cases in Fig. 2(a)]. Although absolute output amplitudes were smaller than target amplitudes due to the effect of skin stiffness, this reduction can be improved by the adjustment of the feedback gain in the PID controller by taking into account such external disturbance, and/or by the application of robust control theory that has better system stability to the effect of external disturbance than a traditional PID control. Taken together, the developed system shows an adequate capacity to produce multi-effector somatosensory perturbations well controlled in amplitude and time. In the current system, we used only two actuators as a first step in the development. Since the control board used in this system is technically compatible with the parallel control of multiple units using a single simulink program in one single PC, it can possibly be easy to extend to the exploitation of several actuators with appropriate synchronization.

We carried out a temporal order judgement test as a pilot example of application to psychophysiological experiments. The results showed that participants were able to identify the temporal order between two somatosensory stimuli [Fig. 2(c)] with a JND around 25 ms. A similar JND for temporal order judgement has already been obtained in previous studies [e.g., around 20 ms by a tapping stimulation to both index fingers, Fujisaki and Nishida (2009)]. This indicates that the developed system has a sufficient capability to run psychophysiological experiments with the somatosensory system. By extending the available patterns of multi-effector somatosensory stimulation, we expect that the developed system could also be applied to other types of experimental paradigms, related to, e.g., directional sensitivity (upward vs downward in one dimension, or upward vs backward in two dimensions) or detection of specific shapes of skin deformation such as square, circle, and so on [e.g., Fig. 2(a)].

The temporal order judgement test also showed an unexpected bias effect related to the fact that the vertical facial skin deformation was perceived earlier than the horizontal one (up to around 20 ms) even though the vertical stretch was actually produced later. A likely interpretation is that the sensitivity for vertical movements may be higher than for horizontal movements, possibly related to the direction of jaw movements in speech. Although further examination is required considering the limited number of participants, this result suggests a potential perspective for further investigation of human somatosensory sensitivity along various dimensions.

One of the main usages of the developed system is to investigate somatosensory perception and the mechanism of somatosensory processing itself not only in speech function but also in general human function. This could involve applying several directions of stimulation on a single site as in the present study, or stimulation at different sites within a single experimental paradigm. While previous experiments have already explored these ideas but using between-subject procedures (Ito *et al.*, 2009; Ito and Ostry, 2012; Ogane *et al.*, 2020), the proposed system allows one to apply within-subject procedures. This may result in a large increase of reliability and statistical power for experimental results and interpretation. The developed system could also be applied in the framework of multisensory integration combining multi-effector somatosensory perturbations with speech auditory and/or visual stimulation as in previous studies (Ito *et al.*, 2009; Ito and Ostry, 2012; Ogane *et al.*, 2020) including EEG study (Ito *et al.*, 2014). This can enhance our ability to efficiently investigate speech perception mechanisms and their interaction with speech production knowledge.

Finally, another set of perspectives concerns motor learning and training. While simple stimulation patterns showed their efficiency to study the effects of motor learning and adaptation in constrained paradigms [e.g., Ito and Ostry (2010)], more flexible paradigms using complicated patterns of somatosensory stimulation can be required for practical situations of training or learning in contexts of education (e.g., foreign language learning) or clinical rehabilitation. The proposed framework can be powerful tools for such application of motor training or learning.

## 6. Conclusion

The proposed framework for the implementation of a multi-directional perturbation system applied to facial skin deformation should enable more realistic and complex stimulation patterns compared to what was done in previous experiments in the field. The system is based on multiple actuators with force control, and can be implemented within a relatively low-cost package. As a first instantiation, we developed a two-directional perturbation system and showed that the system performs well, which enabled us to carry out a pilot psychophysical experiment. This new multi-directional framework could be a powerful tool for further investigations concerning speech production, speech perception, and speech learning, with potential practical applications in speech training.

## Footnotes

^1^ See supplementary material at https://doi.org/10.1121/10.0001942 for the recorded data and analysis programs of the temporal order judgement test.
